# Altered Effective Connectivity of Children and Young Adults With Unilateral Amblyopia: A Resting-State Functional Magnetic Resonance Imaging Study

**DOI:** 10.3389/fnins.2021.657576

**Published:** 2021-07-06

**Authors:** Peishan Dai, Xiaoyan Zhou, Yilin Ou, Tong Xiong, Jinlong Zhang, Zailiang Chen, Beiji Zou, Xin Wei, Ying Wu, Manyi Xiao

**Affiliations:** ^1^School of Computer Science and Engineering, Central South University, Changsha, China; ^2^Hunan Engineering Research Center of Machine Vision and Intelligent Medicine, Central South University, Changsha, China; ^3^Department of Ophthalmology, The Second Xiangya Hospital, Central South University, Changsha, China; ^4^Hunan Clinical Research Center of Ophthalmic Disease, Changsha, China

**Keywords:** resting-state, fMRI, unilateral amblyopia, effective connectivity, granger causality analysis

## Abstract

The altered functional connectivity (FC) in amblyopia has been investigated by many studies, but the specific causality of brain connectivity needs to be explored further to understand the brain activity of amblyopia. We investigated whether the effective connectivity (EC) of children and young adults with amblyopia was altered. The subjects included 16 children and young adults with left eye amblyopia and 17 healthy controls (HCs). The abnormalities between the left/right primary visual cortex (PVC) and the other brain regions were investigated in a voxel-wise manner using the Granger causality analysis (GCA). According to the EC results in the HCs and the distribution of visual pathways, 12 regions of interest (ROIs) were selected to construct an EC network. The alteration of the EC network of the children and young adults with amblyopia was analyzed. In the voxel-wise manner analysis, amblyopia showed significantly decreased EC between the left/right of the PVC and the left middle frontal gyrus/left inferior frontal gyrus compared with the HCs. In the EC network analysis, compared with the HCs, amblyopia showed significantly decreased EC from the left calcarine fissure, posterior cingulate gyrus, left lingual gyrus, right lingual gyrus, and right fusiform gyrus to the right calcarine fissure. Amblyopia also showed significantly decreased EC from the right inferior frontal gyrus and right lingual gyrus to the left superior temporal gyrus compared with the HCs in the EC network analysis. The results may indicate that amblyopia altered the visual feedforward and feedback pathway, and amblyopia may have a greater relevance with the feedback pathway than the feedforward pathway. Amblyopia may also correlate with the feedforward of the third visual pathway.

## Introduction

Amblyopia (also called lazy eye) can be defined as a disorder that is associated with dysfunction of the processing of visual information, and the dysfunction can be detected and evident as reduced recognition visual acuity (Holmes and Clarke, 2006). It usually occurs in just one eye, when there is a breakdown in the working mode of the brain and eyes, and the brain cannot recognize the sight of the lazy eye, resulting in the brain relying more on the other eye^1^. Amblyopia is associated with abnormal visual function in the brain, especially the function of the visual cortex (Huang et al., 2017). It is a developmental visual disease whose visual defects cannot be improved by refractive correction, and the structural abnormalities accompanied by visual impairment are insignificant (Roper-Hall, 2007). It starts in early childhood, which is the critical period of treatment (Tailor et al., 2016), and affects approximately 2%–5% of the general population (Guo et al., 2016).

In general, amblyopia is a neurodevelopmental problem, not an eye organic problem (Li et al., 2012). As functional magnetic resonance imaging (fMRI) can be used to investigate brain activity non-invasively, it has been extensively used to assess visual deficits including amblyopia (Brown et al., 2016). There are two main ways to analyze the brain activity of amblyopia using fMRI data: the regional brain activity analysis [including the amplitude of low-frequency fluctuation (ALFF) (Liang et al., 2016; Min et al., 2018) and the regional homogeneity (ReHo) (Lin et al., 2012; Yang et al., 2019)] and interregional connectivity analysis [including functional connectivity (FC) and effective connectivity (EC)].

Functional connectivity describes the statistical dependence of spatially isolated neuronal events, thereby reflecting the altered interactions among the brain regions in amblyopia. Ding et al. (2013) used resting-state fMRI to study the FC differences between patients with amblyopia and normal controls and found that the cerebellum and the inferior parietal lobule showed altered FC with the primary visual area in individuals with amblyopia. Wang et al. (2014) analyzed the abnormalities of amblyopia patients by both the seed-based FC with the left/right primary visual cortex (PVC) and the network constructed throughout the whole brain and found decreased FC to superior occipital gyrus, lingual gyrus, and several areas in the temporal cortex. Zhou et al. (2015) analyzed the resting-state fMRI of children with strabismus amblyopia and found that the brain FC of patients is lower than the healthy controls (HCs) in the occipital lobe, temporal lobe, posterior cerebellar lobe, parietal lobe, frontal lobe, and cingulate gyrus. Mendola et al. (2018) used resting-state fMRI to study the FC of adult amblyopia in V1, V2, and V3 in a subcortical area manner and found decreased FC within V1, V2, and V3. Lu et al. (2019) used resting-state fMRI to study the FC networks between patients with amblyopia and normal controls and found a decrease of both network functional correlations and local efficiencies in the extra-striate visual networks. Dai et al. (2019) used resting-state fMRI to study the FC networks between patients with amblyopia and normal controls. They found reduced FC in the dorsal and ventral visual pathways. These studies found many altered FCs in or out of the visual pathway, but as different data sets and different data analysis strategies were used, these results are with no high comparability.

Functional connectivity does not reflect the specific causality of brain regions, whereas EC makes up this deficiency. EC depends on the mechanism of the causal effects that generated from the data, which can reflect the specific strength and direction of interaction information in the brain region (Stephan and Friston, 2010). Some researchers began to apply EC to fMRI analysis (Sharaev et al., 2016; Wang et al., 2016; Park et al., 2017; Chen et al., 2018; Ma et al., 2018). EC can be analyzed by various methods. Ohta et al. (2018) explored the causal relationship between the psychosocial aspects of subjective quality of life, symptoms, cognitive functions, and salience network dysfunction in schizophrenia by establishing a structural equation model. Hofmann and Straube (2019) used a dynamic causal model to estimate the EC between BNST and amygdala nuclei and found that there were positive EC between all amygdala nuclei. These studies are model-driven methods that require considerable prior knowledge. In this study, we used Granger causality analysis (GCA), which is a data-driven EC calculation method and can be directly applied to the resting-state data (Liu et al., 2018; Ning et al., 2018; Shi et al., 2019). It has been widely used for time-directed prediction between BOLD fMRI time series to measure the causal effects among brain regions (Jiao et al., 2011).

Few researchers have analyzed altered EC in amblyopia. To the best of our knowledge, only Li et al. (2011) investigated the altered EC of task fMRI of amblyopia and found reduced EC in both feedforward and feedback pathway in the lateral geniculate nucleus and visual cortex when driven by the amblyopic eye. However, this research studied the EC within the lateral geniculate nucleus and visual cortex, which are just part of the visual pathway, with only six amblyopia and there were no HCs. The subjects were a mixture of three types of amblyopia, and they have been receiving different treatments. The cortical impairment in amblyopia is not only limited to the visual cortex but also related to the visual pathway and other complex networks (Wu and Liu, 2017).

In this study, we investigated the altered EC of children and young adults with unilateral amblyopia compared to the HCs using resting-state fMRI. In current researches in amblyopia, the amblyopia group (AMs) usually includes left eye, right eye, and bilateral amblyopia. To reduce the sample interference by the mixture of the left, right, and bilateral eye amblyopia, we chose children and young adults with unilateral amblyopia as the research object. We hypothesized that unilateral amblyopia may affect causal connectivity, which can be measured by the alteration of brain EC, and the brain regions with altered EC may not just be limited to the dorsal and ventral pathways, as there may exist a third visual pathway on the lateral brain surface that is anatomically segregated from the two pathways (Pitcher and Ungerleider, 2020). Our study may reveal some alterations of visual feedforward and feedback pathway of amblyopia.

## Materials and Methods

### Participants

The experiment in this paper was approved by the ethics committee of the Second Xiangya Hospital, Central South University. The HCs had no amblyopia-related diseases, with a corrected visual acuity of ≤0.1 logMAR. All the subjects understood the purpose of this study and signed the written informed consent, and all participants received the same detailed eye examinations. A total of 16 children and young adults with amblyopia and 17 HCs were recruited. We used the thresholds 2 mm translation/2 deg rotation to exclude the subjects with big head movements. As a result, 13 individuals were retained in the amblyopia group (AMs), and 13 individuals were retained in the HCs. All of the subjects had no history of other ocular diseases, surgery, or other treatments. The demographic information of the participants is summarized in Table 1.

**TABLE 1:** Demographic information of subjects.

Subject	Gender	Age	Amblyopic type	Amblyopic eye	CVA (LogMAR)		History of treatment
	
					OD	OS	
Amb 01	F	12	ANA	OS	−0.1	0.7	None
Amb 02	M	5	ANA	OS	0.1	0.4	None
Amb 03	F	14	ANA	OS	0.0	1.0	None
Amb 04	M	8	AMA	OS	−0.1	0.5	None
Amb 05	M	6	ANA; AMA	OS	0.0	1.0	None
Amb 06	M	13	ANA	OS	0.0	0.4	None
Amb 07	M	8	ANA; AMA	OS	0.1	0.7	None
Amb 08	M	10	ANA; AMA	OS	0.0	0.7	None
Amb 09	F	14	ANA; AMA	OS	0.0	0.5	None
Amb 10	M	12	ANA	OS	0.0	1.2	None
Amb 11	F	8	ANA	OS	0.0	0.7	None
Amb 12	M	24	AMA	OS	0.0	0.2	None
Amb 13	M	15	AMA	OS	0.0	0.5	None
Control 01	F	6	None	None	0.2	0.1	None
Control 02	F	14	None	None	0.0	0.0	None
Control 03	F	12	None	None	0.0	0.0	None
Control 04	F	12	None	None	0.0	0.0	None
Control 05	M	9	None	None	0.0	0.0	None
Control 06	F	8	None	None	0.0	0.0	None
Control 07	M	13	None	None	0.0	0.0	None
Control 08	M	14	None	None	0.0	0.0	None
Control 09	F	14	None	None	−0.2	−0.1	None
Control 10	F	11	None	None	0.0	0.0	None
Control 11	M	10	None	None	−0.1	−0.2	None
Control 12	M	7	None	None	0.0	−0.1	None
Control 13	M	10	None	None	−0.2	−0.1	None

*Amb, amblyopia group; F, female; M, male; CVA, corrected visual acuity; OD, oculus dexter; OS, oculus sinister; ANA, anisometropic amblyopia; AMA, ametropic amblyopia.*

### Data Acquisition

The data acquisition was performed with a Philips 3.0-T nuclear magnetic resonance scanner. The subjects were scanned by the MRI scanner in the resting state. All participants were asked to close their eyes, relax their bodies, and have no thinking tasks or external stimuli during the whole scanning process. The lights were dimmed and the participants were asked to wear earmuffs to reduce noise during scanning. The participants’ heads were fixed with foam blocks to reduce head movements during testing. The T1-weighted anatomical data were obtained with the parameters as follows: TE = 2.7 ms, TR = 5.8 ms, FA = 8°, voxel size = 1 mm^3^. The fMRI data were obtained by echo-planar imaging, and the parameters are as follows: TR = 2,000 ms, TE = 30 ms, FA = 90°, number of slices = 36, slice thickness = 4 mm, FOV = 240 mm × 240 mm × 144 mm, and time points = 189, acquisition time = 6 min 18 s.

### Data Processing

The data in this paper were preprocessed based on the DPARSF tool in the SPM8 toolkit^2^ (Yan et al., 2016). The specific preprocessing steps included data format conversion, removal of the first 10 time points, slice timing, head motion correction (Friston 24-parameter model), and spatial normalization to the Montreal Neurological Institute (MNI) Template. The structural image was coregistered to the mean functional image and then the structural image was segmented into gray matter, white matter, and cerebrospinal fluid by using a unified segmentation algorithm (New segment). The EPI images were spatially normalized to the MNI space using the normalization parameters estimated in DARTEL. Nuisance covariates (head motion, white matter signal, and cerebrospinal fluid signal) were regressed out, and then we performed smoothing (4 mm full width at half maximum of Gaussian kernel to decrease the spatial noise), linear trends, and band-pass filtering (0.01 Hz < *f* < 0.08 Hz).

### EC Calculation Using Granger Causality Model

We used REST-GCA version 1.8 (a MatLab toolkit for GCA) (Song et al., 2011; Zang et al., 2012) for EC analysis, using a signed-path coefficient algorithm. REST-GCA using the Granger causality model was used to calculate EC. Granger causality model (Granger, 1969, 2001) can be used to analyze the causality among multiple time series. If we define two time series *X*(*t*) and *Y*(*t*) their autoregressive models are as follows:

(1) $$X\,\left(t\right)=\alpha_{11}\,X\,\left(t-1\right)+\alpha_{12}\,X\,\left(t-2\right)+\cdots +\alpha_{1\,p}\,X\,\left(t-p\right)+\varepsilon_{1}\,\left(t\right).$$

(2) $$Y\,\left(t\right)=\beta_{11}\,Y\,\left(t-1\right)+\beta_{12}\,Y\,\left(t-2\right)+\cdots +\beta_{1\,p}\,Y\,\left(t-p\right)+\varepsilon_{2}\,\left(t\right).$$

The regression models introducing each other are as follows:

(3) $$X\,\left(t\right)=\left[\alpha_{1}\,X\,\left(t-1\right)+\cdots +\alpha_{p}\,X\,\left(t-p\right)\right]+\left[\delta_{1}\,Y\,\left(t-1\right)+\cdots +\delta_{p}\,Y\,\left(t-p\right)\right]+\varepsilon_{3}\,\left(t\right)$$

(4) $$Y\,\left(t\right)=\left[\beta_{1}\,Y\,\left(t-1\right)+\cdots +\beta_{p}\,Y\,\left(t-p\right)\right]+\left[\gamma_{1}\,X\,\left(t-1\right)+\cdots +\gamma_{p}\,X\,\left(t-p\right)\right]+\varepsilon_{4}\,\left(t\right).$$

where ε_*i*_(*t*)*i* = 1, 2, 3, 4 represent prediction residual; α_1*p*_,β_1*p*_,α_*p*_,β_*p*_,δ_*p*_,andγ_*p*_ represent the regression coefficients of the models; and *p* is the order of the model.

If the signed-path coefficient δ_1_, δ_2_,⋯δ_*p*_ is significantly larger or smaller than zero, then*Y*(*t*) significantly Granger cause *X*(*t*) and vice versa, if the signed-path coefficient γ_1_, γ_2_,⋯γ_*p*_ is significantly larger or smaller than zero, then *X*(*t*) significantly Granger causes *Y*(*t*). The positive/negative signed-path coefficients indicate that the activity in one brain region could, respectively, predict the increased/decreased brain activity in another brain region, which may mean excitation/inhibition effects (Zang et al., 2012). The signed-path coefficient value is the value of EC, and the absolute value of EC is expressed by *k* in this study.

### Statistical Analysis of EC

Considering the fact that completely using the data-driven method to calculate EC in a voxel-wise manner will greatly increase the amount of calculation and the difficulty of analysis (for a subject, the EC value should be calculated within more than 100,000 voxels), we use a combination of prior knowledge and data-driven method to calculate EC value. (1) As the PVC is an important brain region for visual information transmission, we selected bilateral PVC as two seed regions to analyze their EC with other brain regions, and compared EC changes between the AMS and the HCs at the group level. (2) Combining the significantly non-zero EC in the HCs (if the HCs is assumed as baseline) and the distribution of visual pathways, we selected 12 regions of interest (ROIs) to analyze the altered EC of these ROIs in amblyopia compared with the HCs. The analysis steps are as follows.

(1) We selected the bilateral PVC as two seed regions each with a radius of 8 mm representing left PVC (PVC.L) and right PVC (PVC.R), respectively. (2) For each subject, the average time series of fMRI data in the seed region of the PVC.L was extracted and calculated its EC with the time series of other brain regions in a voxel-wise manner, which is abbreviated as PVC.L voxel-wise EC. In the same way, we obtained EC between PVC.R and other brain regions, which is abbreviated as PVC.R voxel-wise EC. We called this step voxel-wise EC. (3) We performed a two-sample *t*-test (*P* < 0.001, AlphaSim correction) on the results of voxel-wise EC to analyze the differences in EC between AMs and HCs (the results are shown in section “Results of Significantly Altered EC Between the Two Groups Using Voxel-Wise EC”). (4) We performed a one-sample *t*-test (*P* < 0.05, AlphaSim correction) on the results of voxel-wise EC in the HCs. Combining the results and the distribution of visual pathways, we selected 12 brain regions (MNI coordinates: brain region peak, radius: 8 mm) with significantly altered voxel-wise EC as ROIs. (5) For each subject, the average time series of fMRI data in each ROI was extracted and its EC was calculated with the average time series of the other ROIs in an ROI-wise manner. We called this step ROI-wise EC. (6) Then, we performed a one-sample *t*-test (*P* < 0.05) on the results of ROI-wise EC in the AMs and HCs, respectively (the results are shown in section “EC Network Within the Groups Using ROI-Wise EC”) (7). Last, we performed a two-sample *t*-test (*P* < 0.05) on the results of ROI-wise EC to analyze the altered EC network between the AMs and HCs (the results are shown in section “Comparisons of EC Networks Between the Two Groups Using ROI-Wise EC”).

## Results

### Results of Significantly Altered EC Between the Two Groups Using Voxel-Wise EC

**FIGURE 1:**
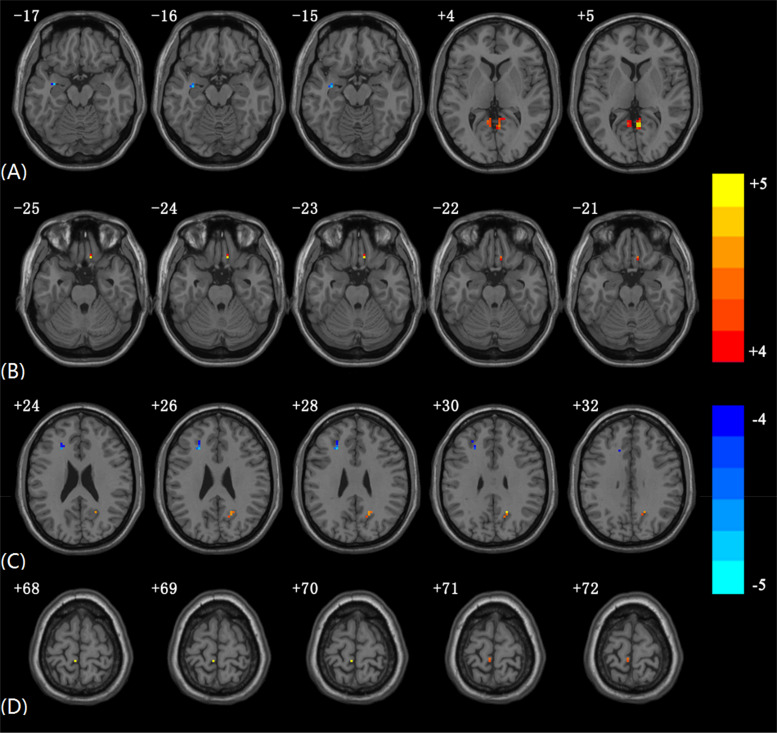
Significantly altered EC in AMs compared to HCs using voxel-wise EC analysis. The color scale represents *T*-values. **(A)** Altered EC from PVC.L to other brain regions in AMs compared to HCs. **(B)** Altered EC from other brain regions to PVC.L in AMs compared to HCs. **(C)** Altered EC from PVC.R to other brain regions in AMs compared to HCs. **(D)** Altered EC from other brain regions to PVC.R in AMs compared to HCs (In each subgraph, only the slices with obvious differences are presented; the subgraphs including slices without significant differences EC are provided in Supplementary Figure 1).

Compared with the HCs, the AMs showed significantly decreased EC from the left PVC to the left hippocampus. Increased EC was found from the left PVC to the left lingual, right lingual, and right precuneus. Amblyopia showed increased EC from the right gyrus rectus to the left PVC (Figure 1 and Table 2). Some individual results of voxel-wise EC are provided in Supplementary Figures 2–5.

**TABLE 2:** Voxel coordinates with the significant EC value between the two groups using voxel-wise EC.

Direction of EC	Brain area	BA	Peak strength	MNI coordinates		
	
				*x*	*y*	*z*
From the PVC.L	HIP.L	20	−4.442	−33	−6	−18
	LING.L	18	4.7339	−9	−51	3
	LING.R	30	6.0667	6	−54	6
	PCUN.R	23	4.3513	18	−57	27
To the PVC.L	REC.R	11	5.4256	9	27	−24
From the PVC.R	MFG.L	10	−4.4512	−30	60	15
	IFG.L	45	−4.5183	−33	42	12
	PUCN.R	23	4.7004	18	−57	30
	MFG.L	48	−4.9637	−24	27	27
	MFG.L	32	−5.3912	−15	24	36
To the PVC.R	PCL.L	4	4.7662	−6	−36	69

*BA, Brodmann’s area; MNI, Montreal Neurological Institute; PVC.L, left primary visual cortex; PVC.R, right primary visual cortex; HIP.L, left hippocampus; LING.L, left lingual gyrus; LING.R, right lingual gyrus; PCUN.R, right precuneus; MFG.L, left middle frontal gyrus; MFG.R, right middle frontal gyrus; REC.R, right gyrus rectus; IFG.L, left inferior frontal gyrus; PCL.L, left paracentral lobule.*

The AMs showed significantly decreased EC from the right PVC to several brain regions, including the left middle frontal gyrus and left inferior frontal gyrus. Increased EC was found from the right PVC to the right precuneus. The AMs showed increased EC from the left paracentral lobule to the right PVC.

### EC in ROIs

According to the EC results in the HCs and the distribution of visual pathways, 12 ROIs (Table 3) were selected to construct the EC network.

**TABLE 3:** Information on the ROIs.

ROI	MNI coordinates			ROI	MNI coordinates		
			
	*x*	*y*	*z*		*x*	*y*	*z*
CAL.L	−12	−72	9	CAL.R	15	−103	3
ACG	6	36	15	PCG	3	−57	9
IFG.L	−36	33	−3	IFG.R	36	33	−3
STG.L	−57	−30	21	STG.R	57	−30	21
FFG.L	−21	−93	−21	FFG.R	21	−93	−21
LING.L	−18	−57	0	LING.R	18	−57	0

*ROI, region of interest; MNI, Montreal Neurological Institute; CAL.L, left calcarine fissure; CAL.R, right calcarine fissure; ACG, anterior cingulate gyrus; PCG, posterior cingulate gyrus; IFG.L, left inferior frontal gyrus; IFG.R, right inferior frontal gyrus; STG.L, left superior temporal gyrus; STG.R, right superior temporal gyrus; FFG.L, left fusiform gyrus; FFG.R, right fusiform gyrus; LING.L, left lingual gyrus; LING.R, right lingual gyrus.*

### EC Network Within the Groups Using ROI-Wise EC

**FIGURE 2:**
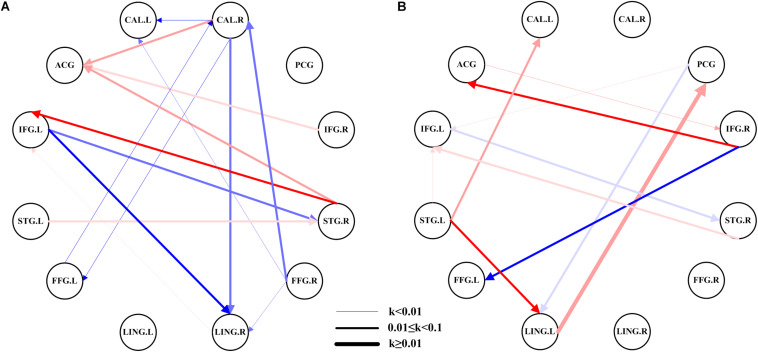
EC network within the groups using ROI-wise EC. **(A)** EC network of ROIs in patients with amblyopia. **(B)** EC network of ROIs in the HCs. The color shades represent significance of EC (0 < *P* < 0.015, 0.015 ≤ *P* ≤ 0.03, and 0.03 < *P* ≤ 0.05); red represents excitation, blue represents inhibition, and the arrow indicates the direction of connection. The thickness of the line represents the connection strength (weak: *k* < 0.01, medium: 0.01 ≤ *k* < 0.1, strong: *k* ≥ 0.1). CAL.L, left calcarine fissure; CAL.R, right calcarine fissure; ACG, anterior cingulate gyrus; PCG, posterior cingulate gyrus; IFG.L, left inferior frontal gyrus; IFG.R, right inferior frontal gyrus; STG.L, left superior temporal gyrus; STG.R, right superior temporal gyrus; FFG.L, left fusiform gyrus; FFG.R, right fusiform gyrus; LING.L, left lingual gyrus; LING.R, right lingual gyrus.

The network of the AMs had 15 pairs of substantial EC (Figure 2A), while the HCs had 11 pairs (Figure 2B). This figure showed considerable differences in the networks between patients with amblyopia and HCs. Most of the EC between ROIs in HCs were excitation, while the patients with amblyopia showed more inhibition. The connectivity of most brain regions was significantly weakened, and some brain effects were lost in patients with amblyopia, such as the EC between the left lingual gyrus and the posterior cingulate gyrus and between the right inferior frontal gyrus and left fusiform gyrus were lost. Besides, the patients with amblyopia also showed significantly altered EC between some brain regions, especially the EC between the right calcarine fissure and some brain regions, which both were inhibited. Some individual results of ROI-wise EC are provided in Supplementary Tables 1–4.

### Comparisons of EC Networks Between the Two Groups Using ROI-Wise EC

**FIGURE 3:**
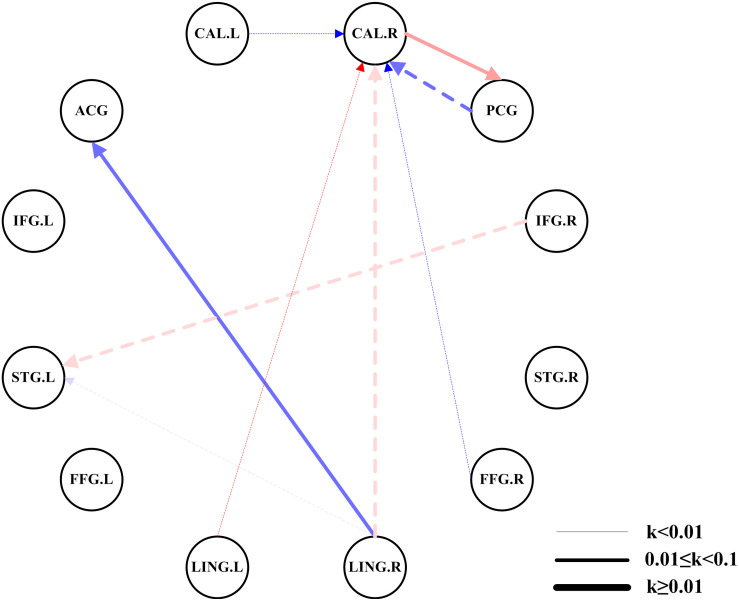
Comparisons of EC networks between the two groups using ROI-wise EC. The color shades represent significance of EC (0 < *P* < 0.015, 0.015 ≤ *P* ≤ 0.03, and 0.03 < *P* ≤ 0.05); red represents excitation, blue represents inhibition, and the arrow indicates the direction of connection. The dotted line indicated a significantly reduced EC in patients with amblyopia. The solid line indicated a significantly increased EC in patients with amblyopia. The thickness of the line represents the connection strength (weak: *k* < 0.01, medium: 0.01 ≤ *k* < 0.1, strong: *k* ≥ 0.1). CAL.L, left calcarine fissure; CAL.R, right calcarine fissure; ACG, anterior cingulate gyrus; PCG, posterior cingulate gyrus; IFG.L, left inferior frontal gyrus; IFG.R, right inferior frontal gyrus; STG.L, left superior temporal gyrus; STG.R, right superior temporal gyrus; FFG.L, left fusiform gyrus; FFG.R, right fusiform gyrus; LING.L, left lingual gyrus; LING.R, right lingual gyrus.

Compared with the HCs, we found significantly altered EC between the two networks in the AMs (Figure 3). The AMs showed reduced EC from the left calcarine fissure, posterior cingulate gyrus, left lingual gyrus, right lingual gyrus, and right fusiform gyrus to the right calcarine fissure, and from the right inferior frontal gyrus and right lingual gyrus to the left superior temporal gyrus. The AMs also showed significantly increased EC from the right lingual gyrus to the anterior cingulate gyrus and from the right calcarine fissure to the posterior cingulate gyrus.

## Discussion

To our knowledge, this work is the first to examine the altered EC of children and young adults with unilateral amblyopia under resting conditions. We found decreased EC in patients with amblyopia by using GCA.

### The Altered EC of Voxel-Wise EC Analysis

We found significantly decreased EC between the left/right of PVC and the left middle frontal gyrus/left inferior frontal gyrus in amblyopia. Both the middle frontal gyrus and inferior frontal gyrus belong to the prefrontal cortex, which plays a significant role in the perception, memory, and regulation of visual information (Majaj et al., 2007). The primate visual system mainly includes the dorsal and ventral pathways. The ventral pathway deals with conscious perception, and the dorsal pathway processes visual information and guides action without accompanying conscious knowledge (Fang and He, 2005). The PVC and prefrontal cortex could be considered as the two ends of the bidirectional (feedforward and feedback pathway) visual pathway. The bidirectional decreased EC between the PVC and prefrontal cortex may indicate that amblyopia altered the visual feedforward and feedback pathway. Ding et al. (2013) also found an FC alteration between the frontal lobe and the PVC in anisometropic amblyopia. Their results conformed to the studies of our EC. Besides, the causal relationship between the PVC and the frontal lobe might contribute to the further study of the neurological mechanism.

We also found decreased EC from the PVC.L to the left hippocampus in patients with amblyopia. The hippocampus is located in the inner region of the temporal lobe and forms part of the limbic system. It plays a significant role in the central nervous system, situational memory, regulation, and learning (Ranganath et al., 2004). Decreased EC may indicate the decreased information transfer to the left hippocampus, but to our knowledge, we found no report about the correlation between hippocampus and amblyopia.

For the feedforward direction from the PVC, we found significantly increased EC from the PVC.L to the left lingual gyrus, right lingual gyrus, and right precuneus, and from PVC.R to the right precuneus in amblyopia. The increased EC may reflect that for the left eye amblyopia, the brain function associated with the left eye may be suppressed, while the brain function associated with the right healthy eye may be increased to form a near-normal visual perception, which may increase the EC associated with the right healthy eye, showing the compensatory plasticity (Lazzouni and Lepore, 2014). Huang and Zhou (2017) found that the ReHo in the lingual gyrus of amblyopia increased. Liang et al. (2016) found that children with amblyopia mainly exhibit increased ALFF in the right precuneus. The EC analysis is consistent with the findings of these studies.

For the feedback direction to PVC, we found significantly increased EC from the right gyrus rectus to the PVC.L and from the left paracentral lobule to the PVC.R. The right gyrus rectus is located at the medial most margin of the inferior surface of the frontal lobe, and it plays an important role in the optic chiasm (Smith et al., 2017). The left paracentral lobule is on the medial surface of the hemisphere and is the continuation of the precentral and postcentral gyrus. The postcentral gyrus is an important brain area of somatosensory function (Ebeling and Steinmetz, 1995). Lin et al. (2012) found increased ReHo in the left paracentral lobule in patients with anisometropic amblyopia. The EC analysis is consistent with the finding of this study. The increased EC from the left paracentral lobule to PVC.R may indicate compensatory plasticity in amblyopia.

### The Altered EC of ROI-Wise EC Analysis

Comparing EC network differences between the two groups, we found significantly reduced EC from the left calcarine fissure, posterior cingulate gyrus, left lingual gyrus, right lingual gyrus, and right fusiform gyrus to the right calcarine fissure in amblyopia. The calcarine sulcus is where the PVC (V1) is concentrated, and the PVC receives the nerve impulses from the optic nerves and then transmits information to two primary pathways, called the ventral stream and the dorsal stream (Bitar et al., 2016). The significantly reduced EC to the right calcarine fissure may indicate that amblyopia may correlate to the reduced feedback of visual information transmission. These results are consistent with the findings of the following researches. Ding et al. (2013) found significantly decreased FC of the PVC and lingual gyrus, the conjunction area of the posterior cingulate cortex, and the precuneus in mixed (anisometropic and strabismic) amblyopia. Huang et al. (2016) using ReHo examined subjects with strabismic amblyopia and found increased ReHo values in the fusiform gyrus, right lingual gyrus, and bilateral cingulate gyrus.

We also found reduced EC from the right inferior frontal gyrus and right lingual gyrus to the left superior temporal gyrus. In a recent study, David Pitcher (Pitcher and Ungerleider, 2020) found evidence for a third visual pathway specialized for social perception, which begins in the PVC (V1) and projects into the posterior banks of the superior temporal sulcus, so the reduced EC to the left superior temporal gyrus indicates that amblyopia may correlate with the feedforward of the third visual pathway. The results are consistent with the findings of the alteration of ReHo (Lin et al., 2012) and the ALFF (Tang et al., 2017) in the left superior temporal gyrus.

Amblyopia had significantly increased EC from the right lingual gyrus/right calcarine fissure to the anterior cingulate gyrus/posterior cingulate gyrus, which belongs to the cingulate gyrus, and this may be due to the visual compensatory mechanism of children and young adults with monocular amblyopia.

The present study has several limitations. (1) The main limitation of our study was the sample size. Given the difficulty of recruitment and the poor controllability of the test data of children’s test subjects, the actual number of subjects studied in the experiment was small, which may reduce the reliability of the statistical results. The patients also all possessed left eye amblyopia. If another group of subjects with right eye amblyopia is added, then the comparative analysis will be comprehensive. In a further study, a stringent threshold for exploratory analysis can be used. (2) In the ROI-wise EC analysis, we selected ROIs based on the analysis of control data and the distribution of visual pathways, which may lead to a bias toward finding between-group differences, and we will conduct a comprehensive analysis by including the AMs in a future study.

## Conclusion

We investigated the brain function causality of children and young adults with unilateral amblyopia through a comparative analysis of rs-fMRI data. The bidirectional decreased EC between the PVC and prefrontal cortex may indicate that amblyopia altered the visual feedforward and feedback pathway. The decreased effective connectivities in ROIs network are most feedback from other visual-related regions to the right calcarine fissure. It may indicate that amblyopia has an imbalanced relationship with the feedforward and the feedback visual pathway, and it may have a greater relevance with the feedback pathway. The significantly decreased EC to the left superior temporal gyrus in the regions of interest network indicates that amblyopia may correlate with the feedforward of the third visual pathway.

## Data Availability Statement

The raw data supporting the conclusions of this article will be made available by the authors, without undue reservation.

## Ethics Statement

The studies involving human participants were reviewed and approved by the Ethics Committee of the Second Xiangya Hospital, Central South University. Written informed consent to participate in this study was provided by the participants’ legal guardian/next of kin.

## Author Contributions

PD: methodology, conceptualization, formal analysis, funding acquisition, investigation, project administration, supervision, validation, writing—original draft, and writing—review and editing. XZ: methodology, software, conceptualization, data curation, formal analysis, visualization, investigation, writing—original draft, and writing—review and editing. YO, TX, ZC, YW, and JZ: formal analysis and investigation. BZ: investigation and project administration. XW: data curation, funding acquisition, investigation, resources, and validation. MX: data curation, funding acquisition, resources, supervision, and writing—review and editing. All authors contributed to the article and approved the submitted version.

## Conflict of Interest

The authors declare that the research was conducted in the absence of any commercial or financial relationships that could be construed as a potential conflict of interest.

## Footnotes

1 https://www.nei.nih.gov/learn-about-eye-health/eye-conditions-and-diseases/amblyopia-lazy-eye

2 http://rfmri.org/DPARSF
