# Supplementary material for: Altered Effective Connectivity of Children and Young Adults With Unilateral Amblyopia: A Resting-State Functional Magnetic Resonance Imaging Study
Source: Front Neurosci. 2021 Jul 6;15:657576. doi: 10.3389/fnins.2021.657576 (PMC8290343; doi:10.3389/fnins.2021.657576)
Supplement: Supplementary file 1 [file Data_Sheet_1.zip › Supplementary Material/Individual voxel-wise EC/Individual voxel-wise EC.docx]

Supplementary Material

**
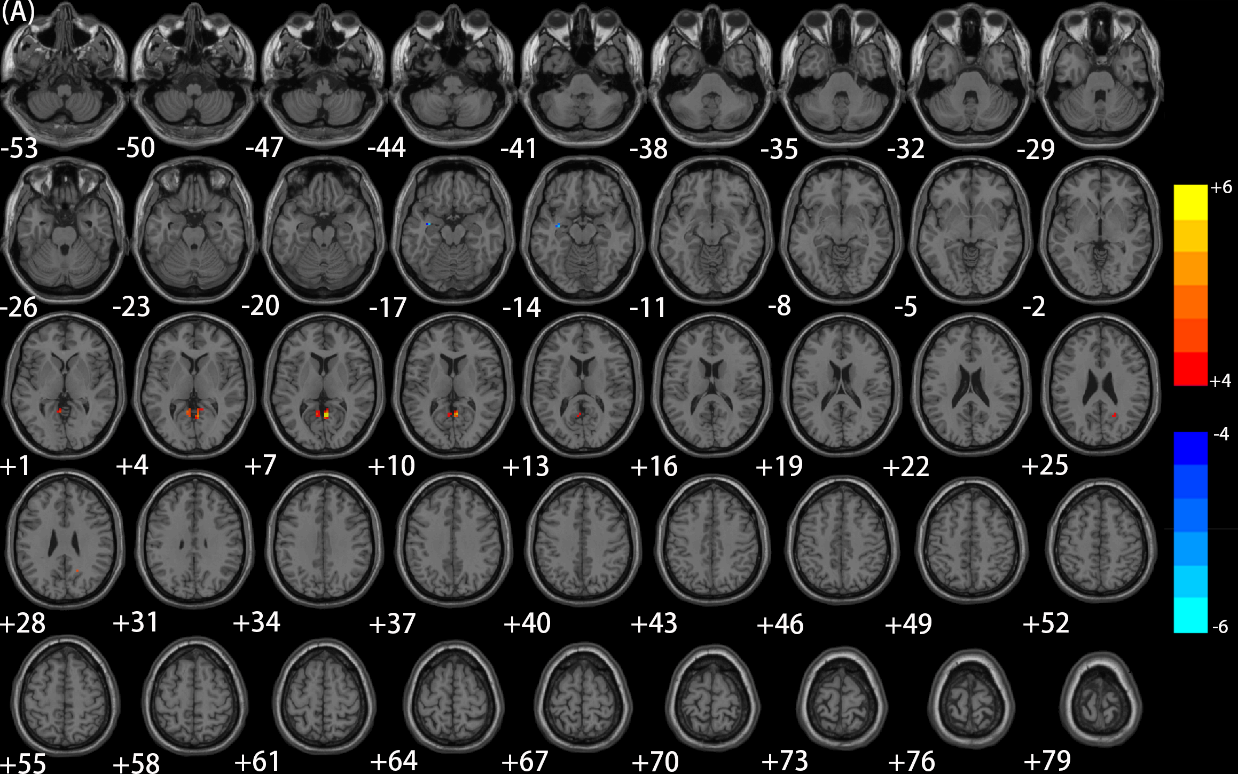
**

**
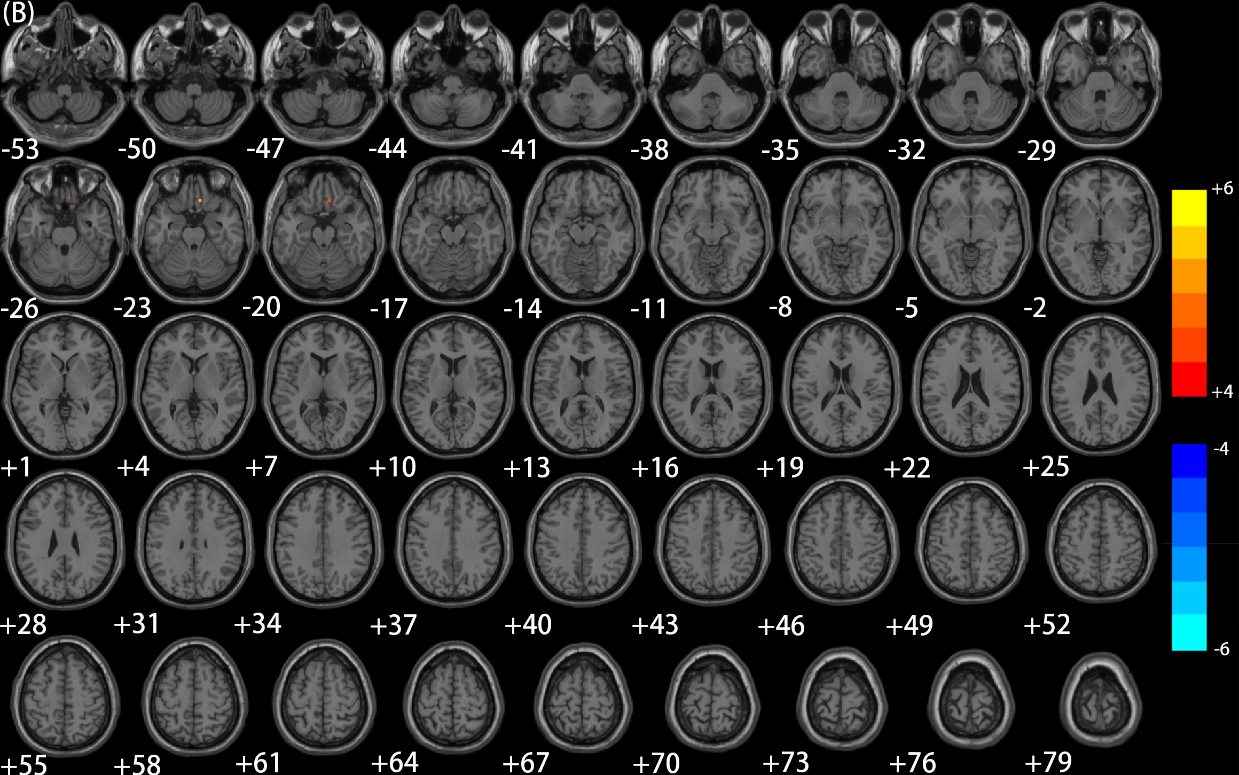
**


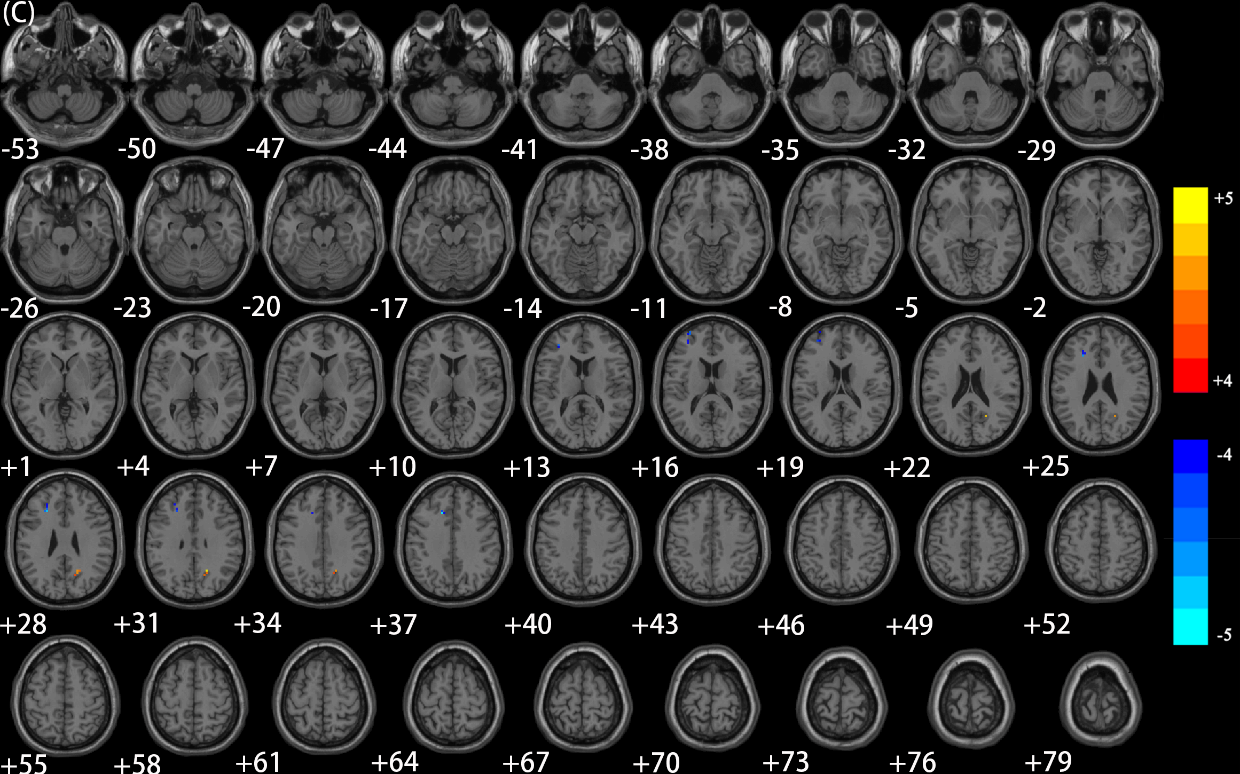


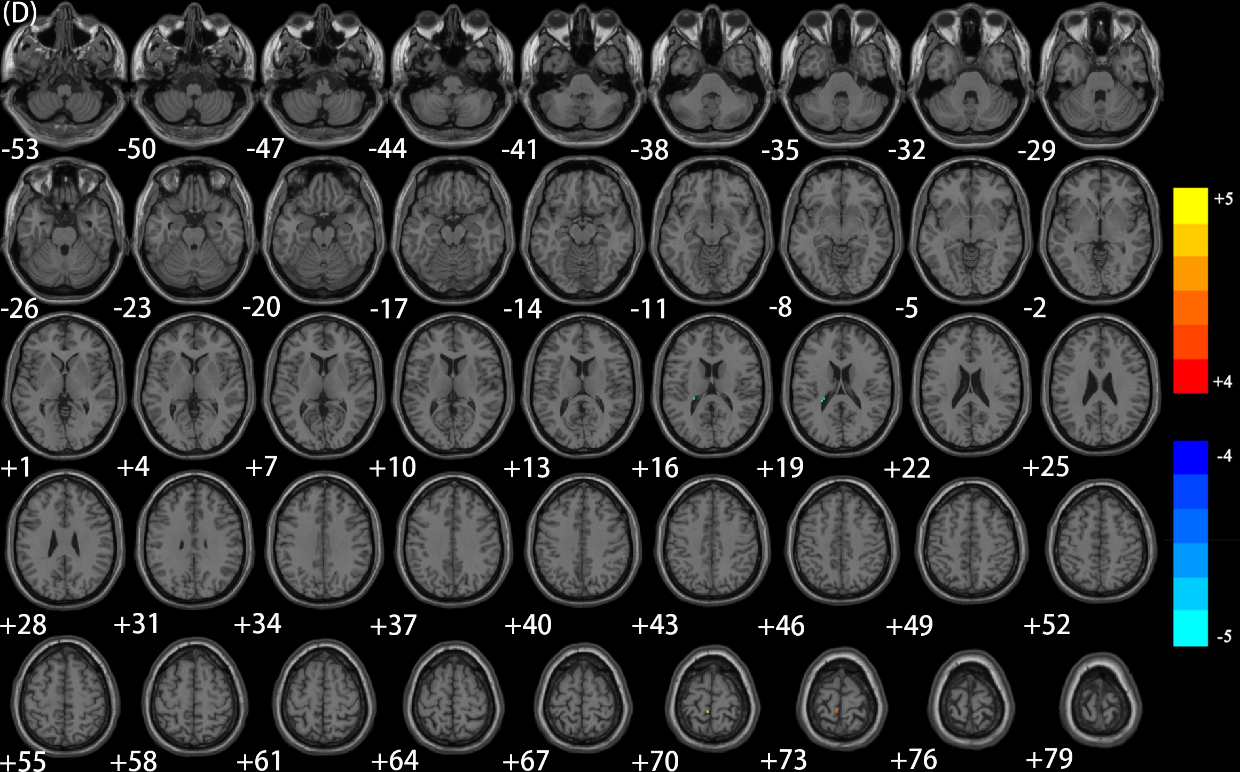


**Figure S1.** Significantly altered EC in AMs compared to HCs using voxel-wise EC analysis. The color scale represents T values. **(A)** Altered EC from PVC.L to other brain regions in AMs compared to HCs. **(B)** Altered EC from other brain regions to PVC.L in AMs compared to HCs. **(C)** Altered EC from PVC.R to other brain regions in AMs compared to HCs. **(D)** Altered EC from other brain regions to PVC.R in AMs compared to HCs.


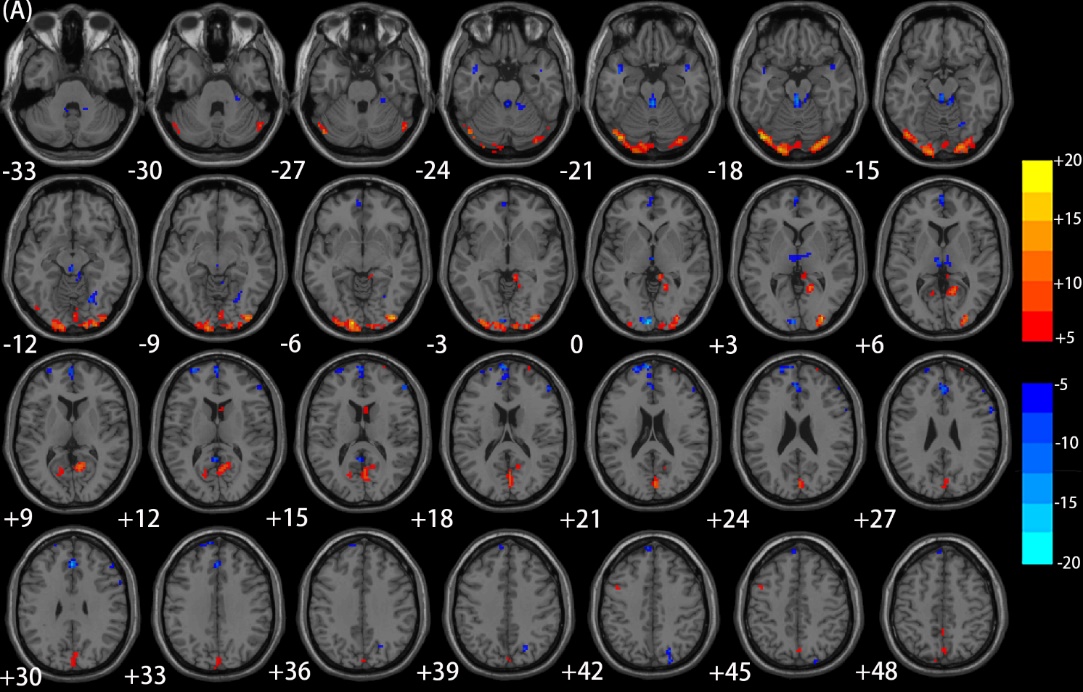


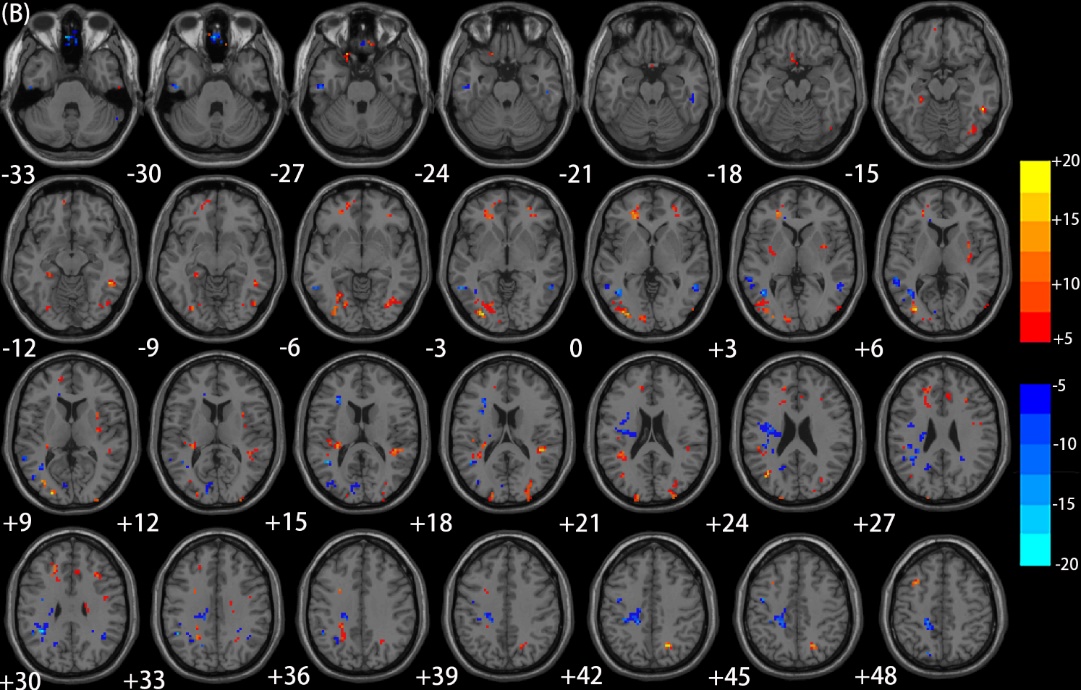


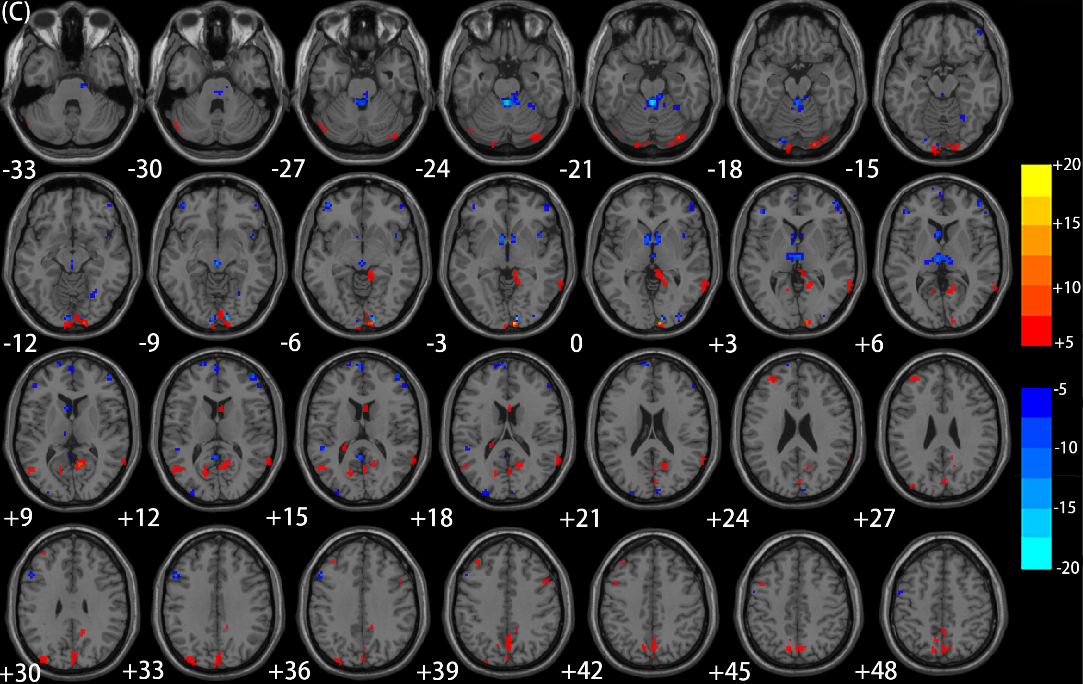


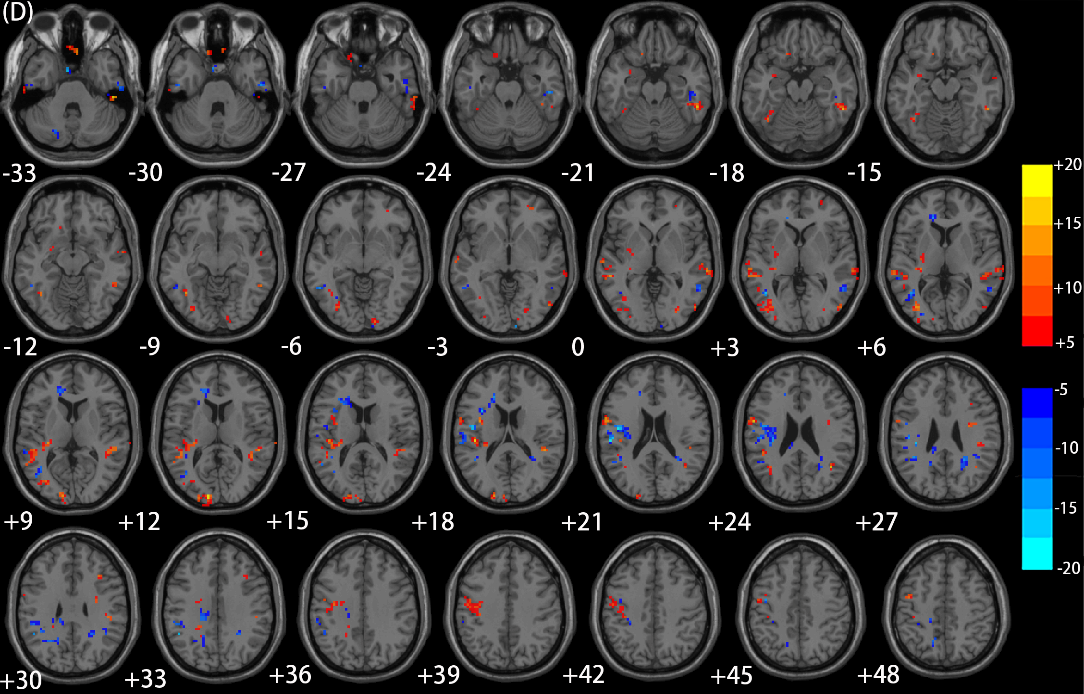


**Figure S2.** Voxel-wise EC between the PVC and other brain regions in a child with amblyopia. The color scale represents EC values (the absolute value of EC is greater than or equal to 5). (A) EC from PVC.L to other brain regions. (B) EC from other brain regions to PVC.L. (C) EC from PVC.R to other brain regions. (D) EC from other brain regions to PVC.R.


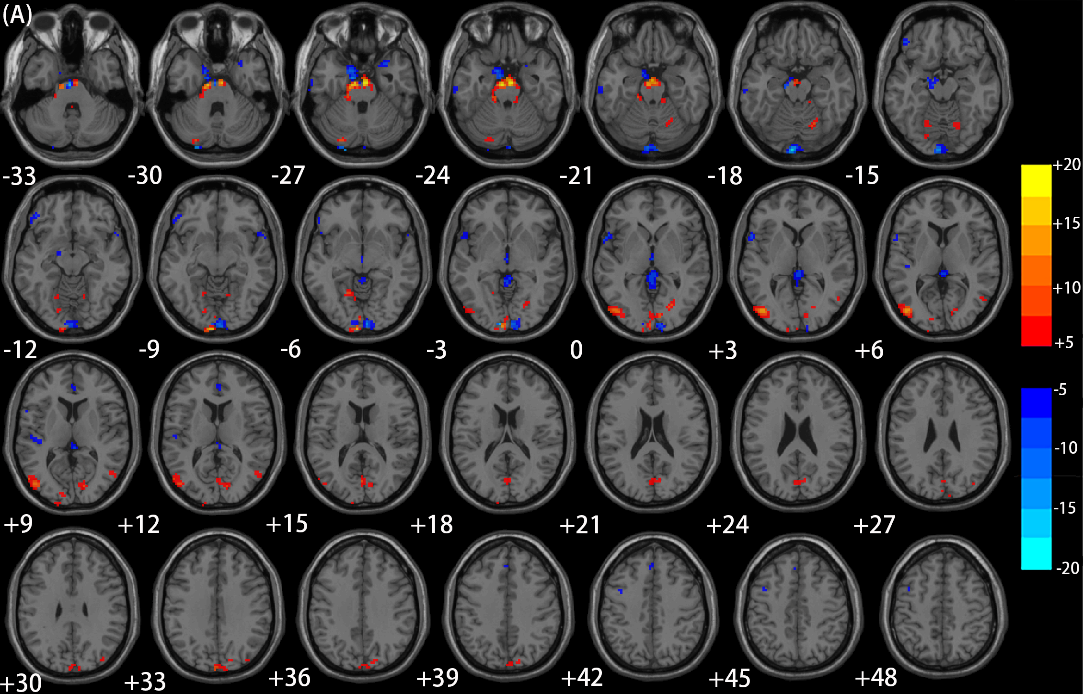


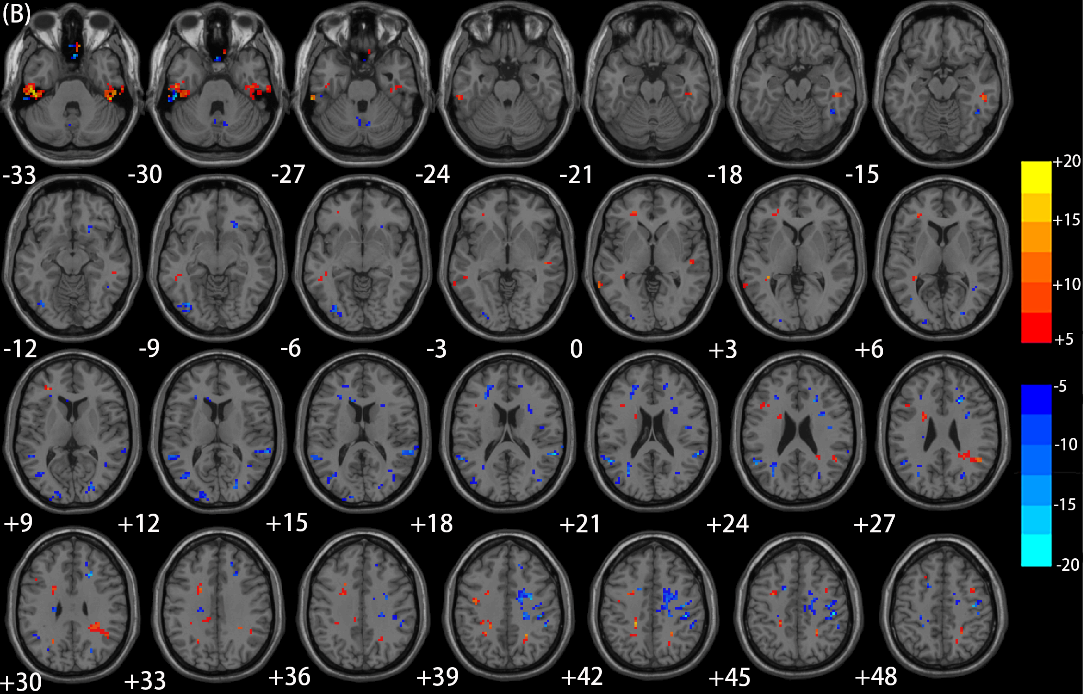


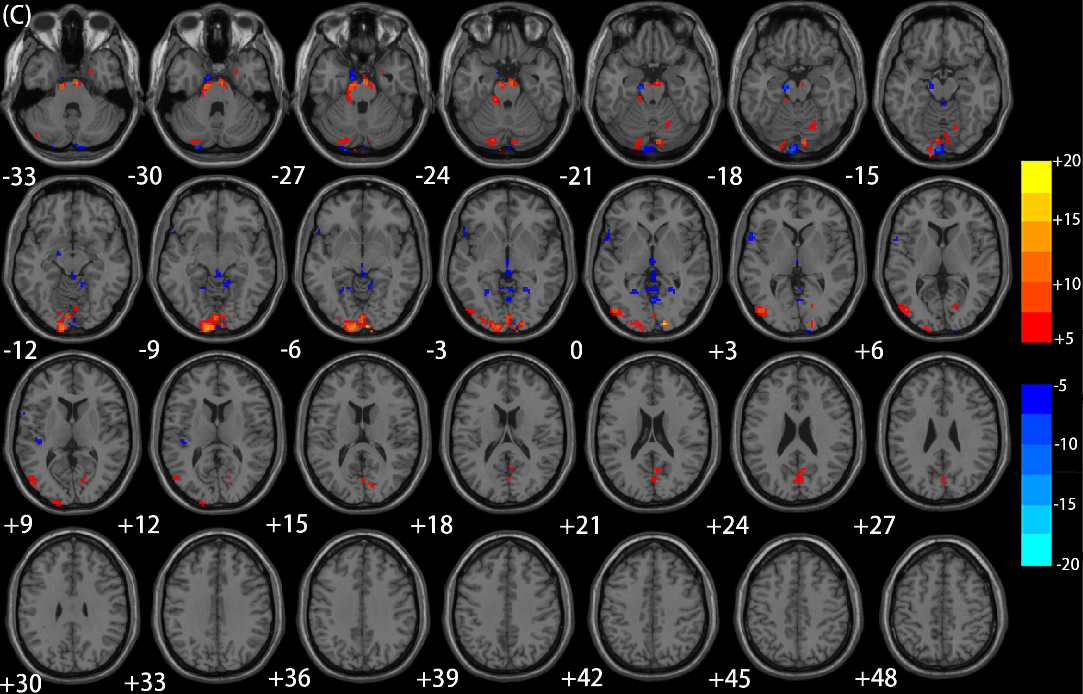


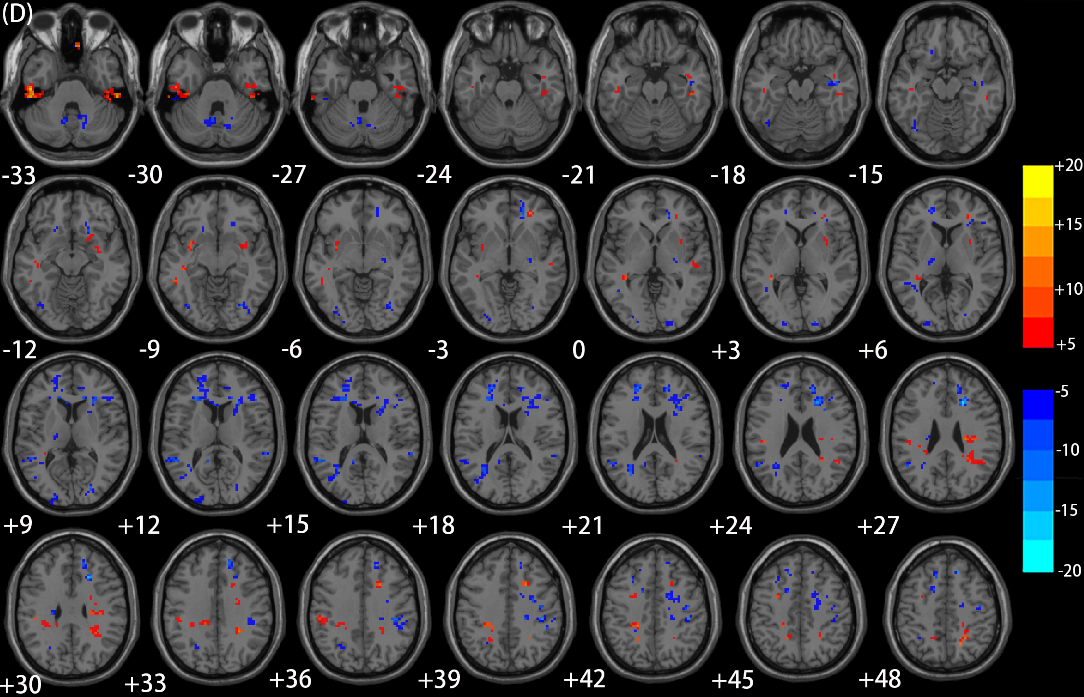


**Figure S3.** Voxel-wise EC between the PVC and other brain regions in a HC. The color scale represents EC values (the absolute value of EC is greater than or equal to 5). (A) EC from PVC.L to other brain regions. (B) EC from other brain regions to PVC.L. (C) EC from PVC.R to other brain regions. (D) EC from other brain regions to PVC.R.


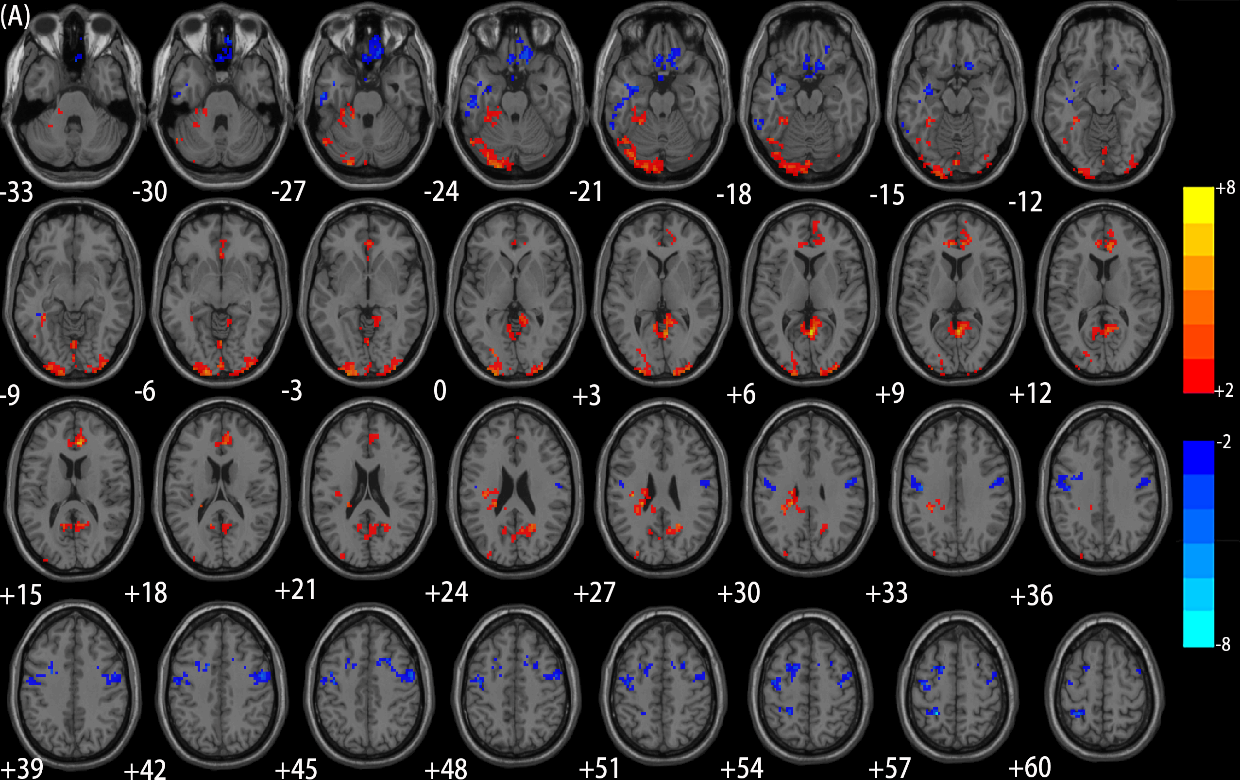


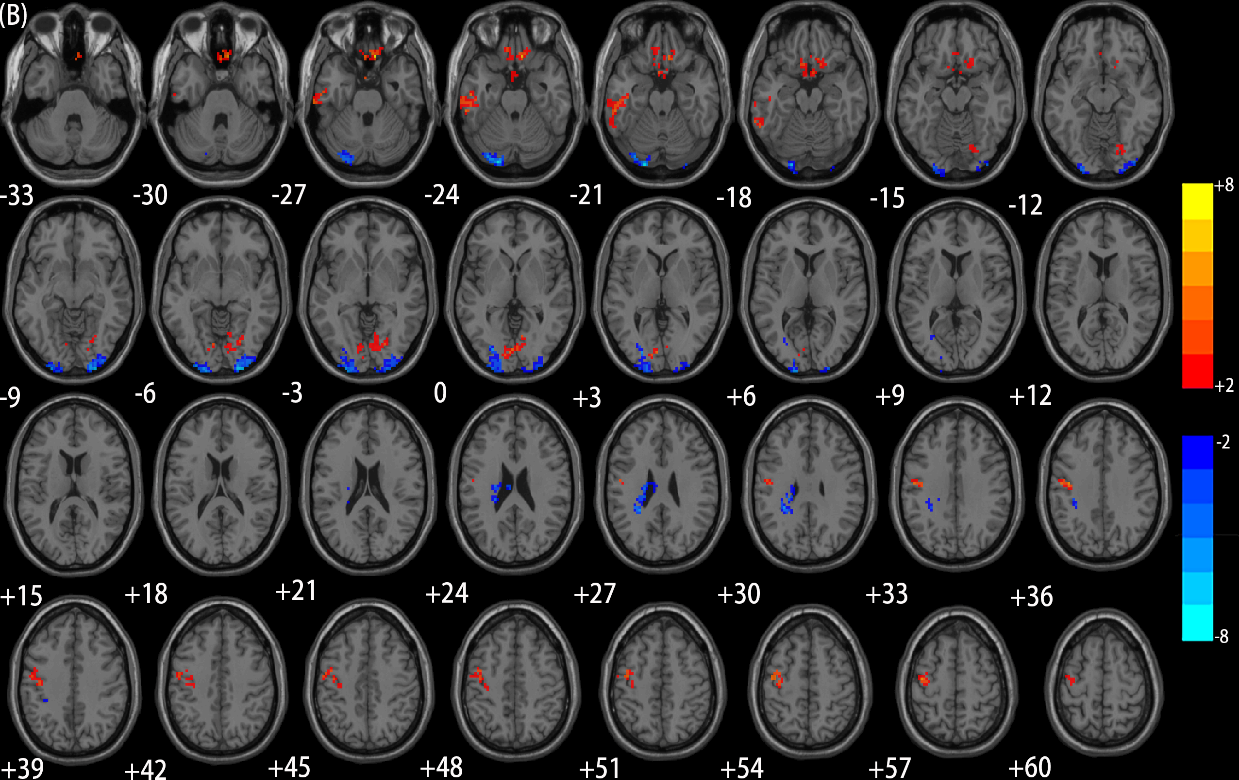


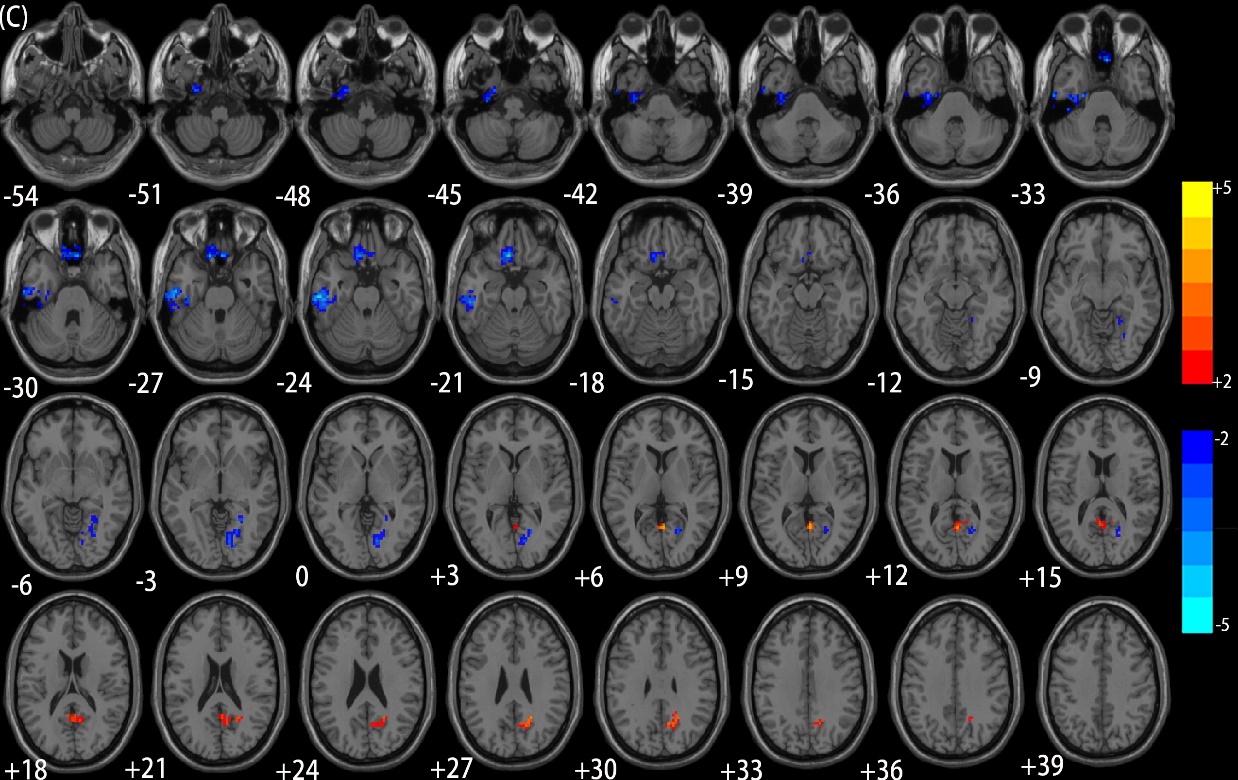


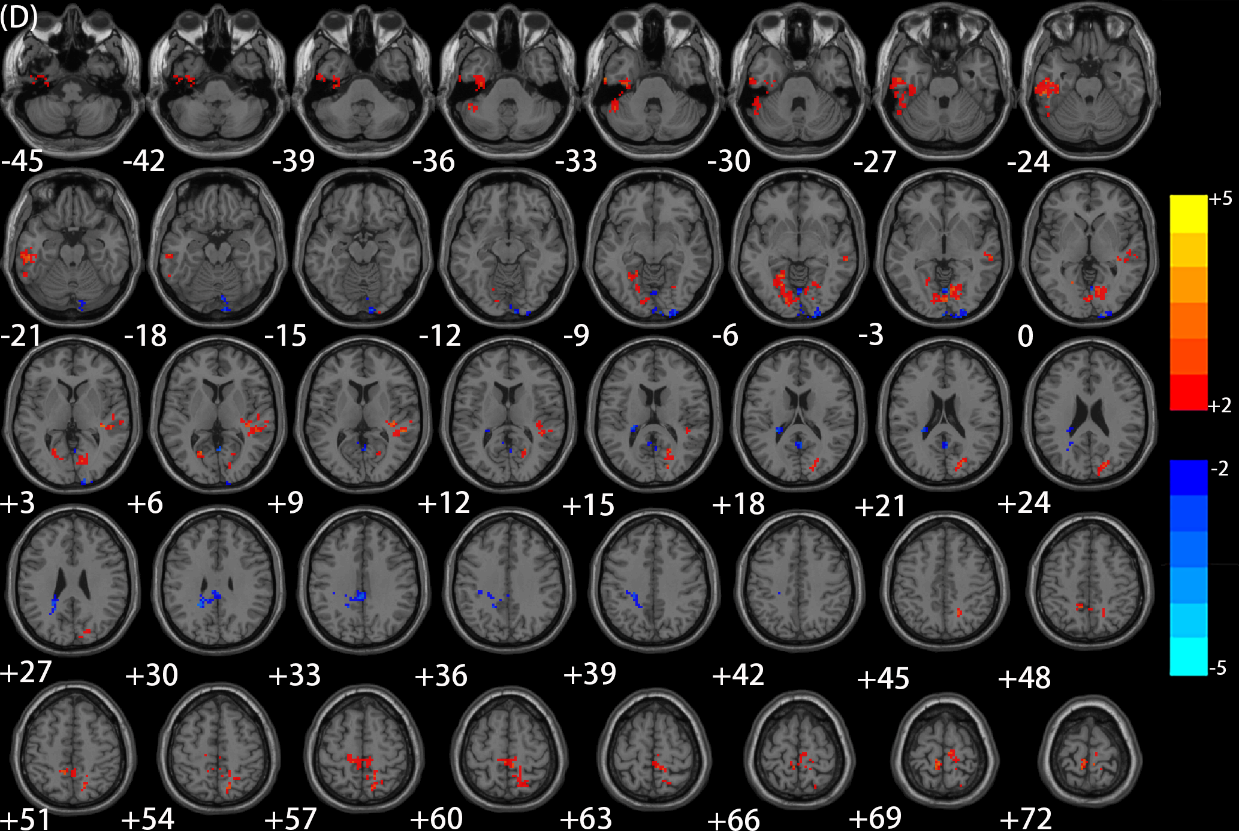


**Figure S4.** Voxel-wise EC between the PVC and other brain regions in the amblyopia group after one-sample t-test. The color scale represents T values. (A) EC from PVC.L to other brain regions. (B) EC from other brain regions to PVC.L. (C) EC from PVC.R to other brain regions. (D) EC from other brain regions to PVC.R.


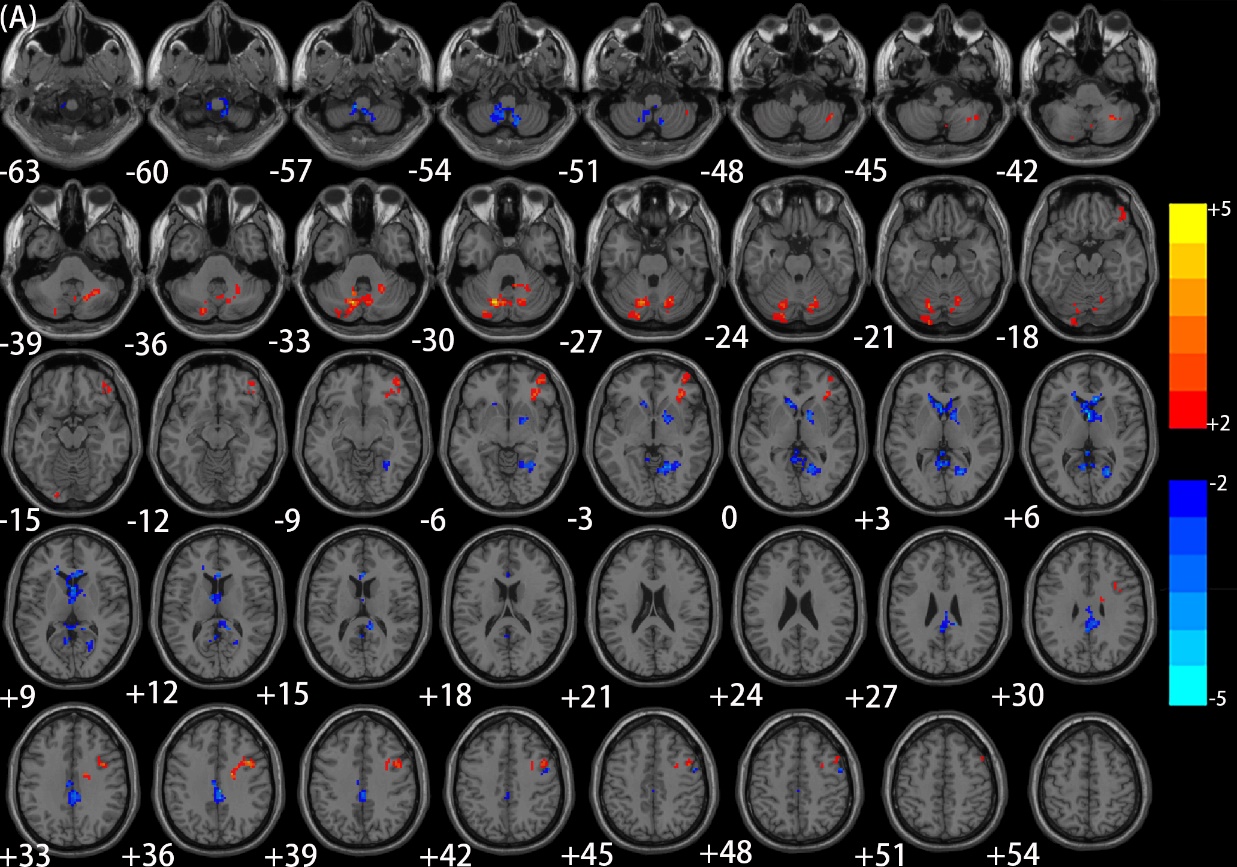


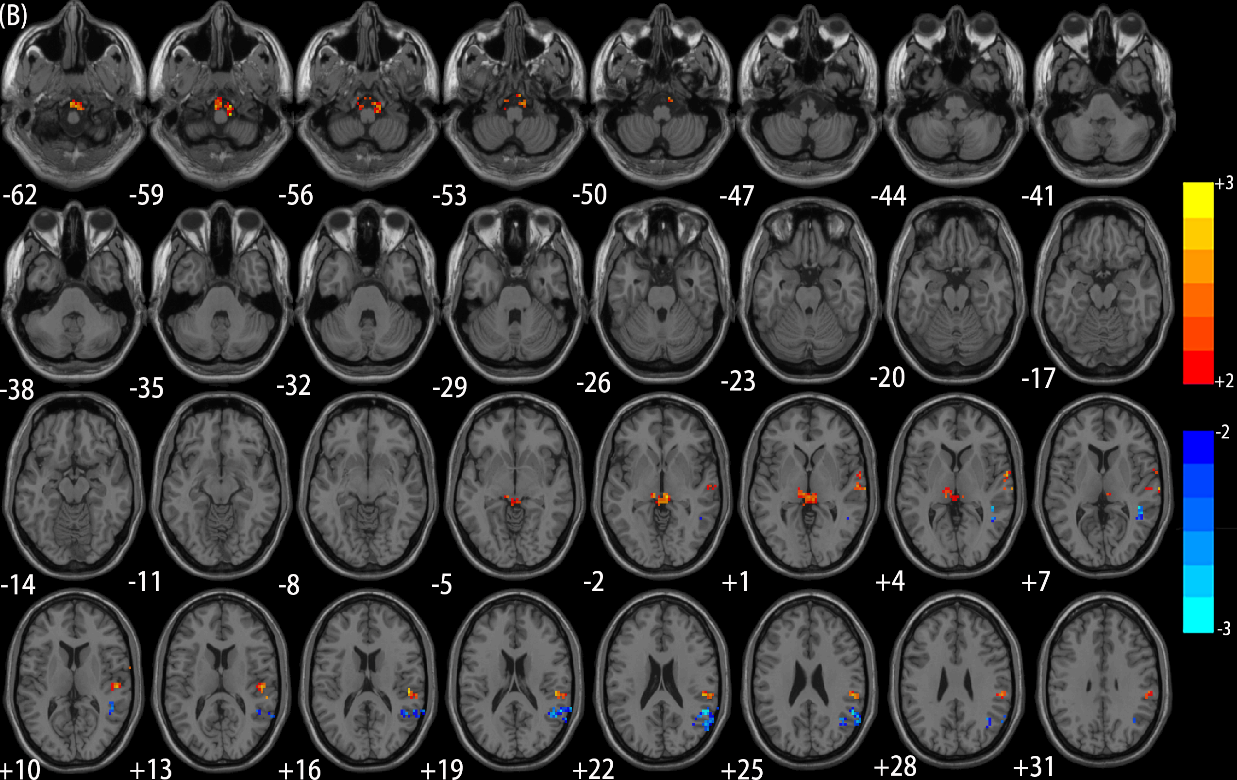


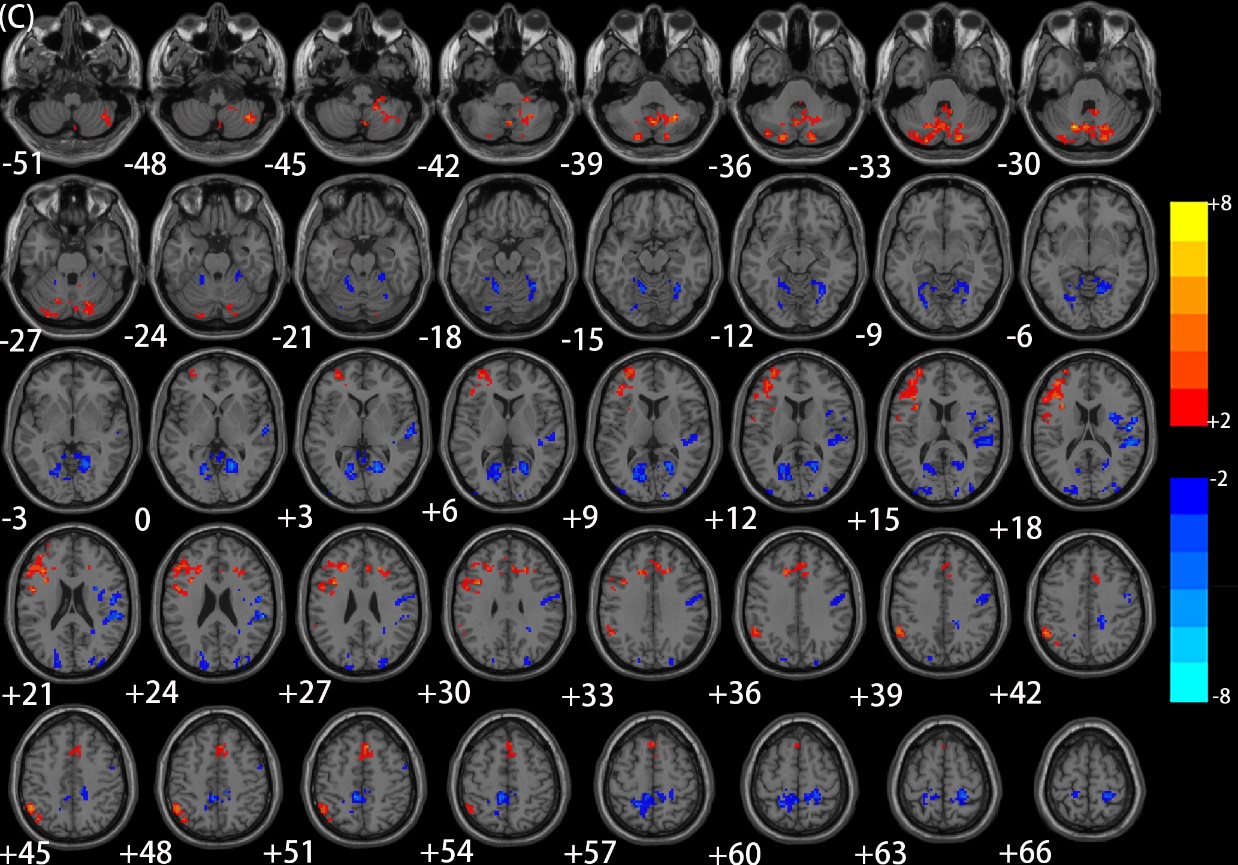


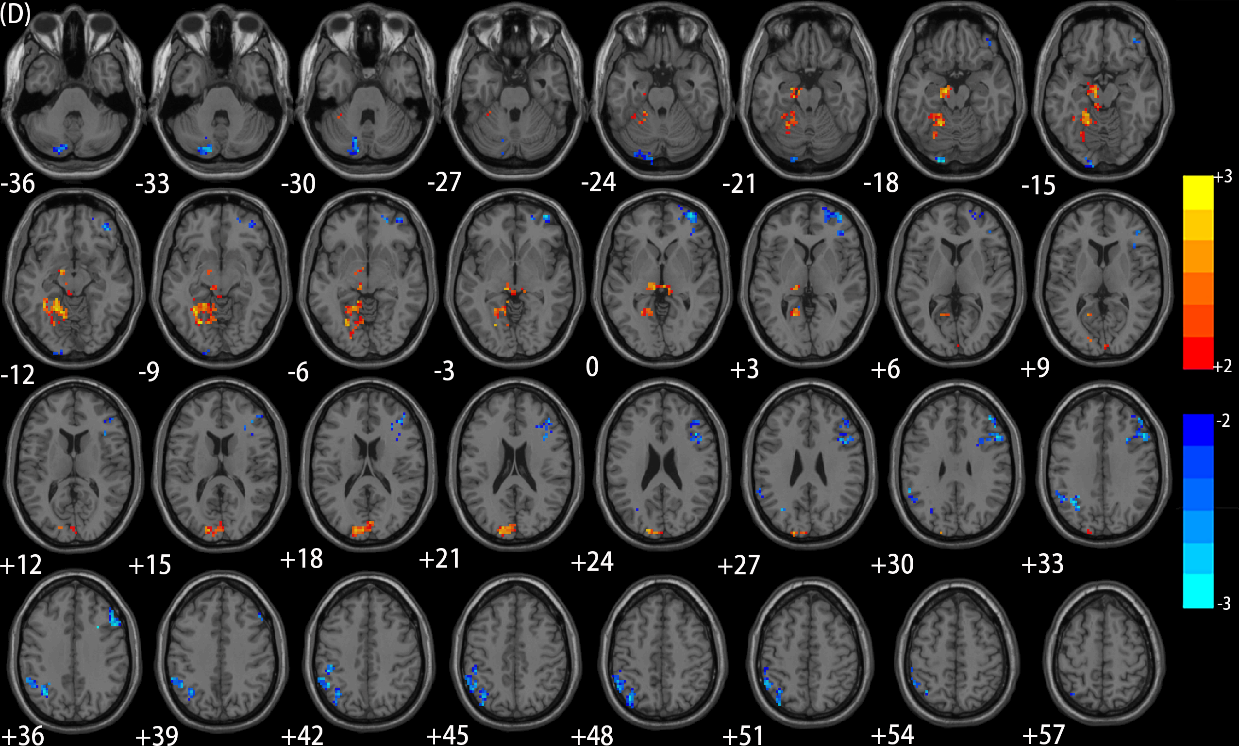


**Figure S5.** Voxel-wise EC between the PVC and other brain regions in the HCs after one-sample t-test. The color scale represents T values. (A) EC from PVC.L to other brain regions. (B) EC from other brain regions to PVC.L. (C) EC from PVC.R to other brain regions. (D) EC from other brain regions to PVC.R.
